# Advances in the design and development of SARS-CoV-2 vaccines

**DOI:** 10.1186/s40779-021-00360-1

**Published:** 2021-12-16

**Authors:** Xue-Liang Peng, Ji-Si-Yu Cheng, Hai-Lun Gong, Meng-Di Yuan, Xiao-Hong Zhao, Zibiao Li, Dai-Xu Wei

**Affiliations:** 1 Key Laboratory of Resource Biology and Biotechnology in Western China, Ministry of Education, School of Medicine, Department of Life Sciences and Medicine, Northwest University, Xi’an, 710069 China; 2 Institute of Materials Research and Engineering, A*STAR (Agency for Science, Technology and Research), 2 Fusionopolis Way, Innovis, #08-03, Singapore, 138634 Singapore

**Keywords:** Severe acute respiratory syndrome coronavirus 2 (SARS-CoV-2), Coronavirus disease 2019 (COVID-19), Vaccine, Synthetic biology, Nanoscience

## Abstract

Since the end of 2019, coronavirus disease 2019 (COVID-19) caused by severe acute respiratory syndrome coronavirus 2 (SARS-CoV-2) has spread worldwide. The RNA genome of SARS-CoV-2, which is highly infectious and prone to rapid mutation, encodes both structural and nonstructural proteins. Vaccination is currently the only effective method to prevent COVID-19, and structural proteins are critical targets for vaccine development. Currently, many vaccines are in clinical trials or are already on the market. This review highlights ongoing advances in the design of prophylactic or therapeutic vaccines against COVID-19, including viral vector vaccines, DNA vaccines, RNA vaccines, live-attenuated vaccines, inactivated virus vaccines, recombinant protein vaccines and bionic nanoparticle vaccines. In addition to traditional inactivated virus vaccines, some novel vaccines based on viral vectors, nanoscience and synthetic biology also play important roles in combating COVID-19. However, many challenges persist in ongoing clinical trials.

## Introduction

The coronavirus disease 2019 (COVID-19) pandemic caused by the new severe acute respiratory syndrome coronavirus 2 (SARS-CoV-2) is an ongoing crisis of grave global concern. More than 4.92 million people had died of COVID-19 by October 20, 2021. The World Health Organization (WHO) announced that the new coronavirus pneumonia epidemic is listed as a Public Health Emergency of International Concern (PHEIC). The infectivity of the original emergent SARS-CoV-2 strain was close to or slightly higher than that of severe acute respiratory syndrome coronavirus 1 (SARS-CoV-1), and it is highly antigenically and genetically similar to SARS-CoV-1 [1]. The whole world must quickly cut the transmission route and adopt effective prevention and control measures to prevent the further spread of SARS-CoV-2. The law of the People's Republic of China on the prevention and control of infectious diseases states, “The state implements a policy of prevention primarily for the prevention and control of infectious diseases.” Since the outbreak of the virus at the end of 2019, it has exerted severe negative effects on human health and the economy [2]. In December 2019, many cases of pneumonia with an unknown etiology were recorded [3]. Scientists announced preliminary data on the identified pathogen on January 8, 2020 and published the whole sequence of the virus genome [4]. The National Pathogen Microbiological Resource Bank at the Chinese Center for Disease Control and Prevention announced the information and electron micrographs of the first virus strains on January 24, 2020. On February 11, 2020, the International Committee on Taxonomy of Viruses (ICTV) named the new coronavirus SARS-CoV-2. On the same day, the World Health Organization named the disease caused by the virus COVID-19 [5]. As of 12:14 Beijing time on October 20, 2021, health authorities worldwide had reported more than 242.38 million confirmed cases and more than 4.92 million deaths, and these numbers are expected to increase further.

Although coronaviruses are often associated with acute respiratory infections in humans, their ability to infect multiple host species makes them complex pathogens. Due to the high prevalence and wide circulation of coronaviruses, the genetic diversity and frequent recombination of their genomes, and frequent interactions between humans and animals, new coronaviruses are likely to emerge periodically in the human population via zoonotic sources [6]. A quick understanding of the structure and characteristics of SARS-CoV-2, as well as the clinical features of COVID-19, is necessary to expedite vaccine research and drug development. In terms of treatment and prevention, scientists are facing great challenges in the process of developing vaccines and drugs because of the variability of SARS-CoV-2, which will necessitate long-term research and development of specific vaccines and drugs against the virus. In addition, SARS-CoV-2 has intermediate to high infectivity. As a result, scientists not only must consider the safety and efficacy of the vaccine but also protective measures for personnel during the research and development process, increasing the challenges associated with the whole enterprise. To date, a global vaccine development strategy based on inactivated virus vaccines, recombinant protein vaccines, recombinant viral vector vaccines, nucleic acid vaccines (e.g., mRNA vaccines and DNA vaccines) and live attenuated vaccines has been launched, and some vaccines have already completed phase III clinical trials and are on the market.

In this paper, various types of vaccines are reviewed, based on which the concept of a bionic simulated virus vaccine is proposed and a new strategy of vaccine development is considered. Bionic technology has been widely used in biology and medicine [7, 8]. Studies have revealed that bionic nanoparticles potentially represent promising mucosal adjuvants for “universal” influenza vaccines, which has made biomimetic technology more mature and provided broad application prospects. In vaccine development, biomedical nanomaterials have been used to simulate the whole virus structure, including its infection process, and efficiently trigger antibody production. Additionally, biomedical nanomaterials have desirable biocompatibility [9, 10]. Thus, the use of this strategy to develop vaccines may be a good choice.

## SARS-CoV-2

### Overview of SARS-CoV-2

SARS-CoV-2 is the seventh known coronavirus that infects humans and causes disease [11]. According to a genomic analysis, SARS-CoV-2 falls within the B subgroup of the *Betacoronavirus* genus, which causes lower respiratory tract infections and pneumonia in humans and has a high mutation rate, alternative splicing, and overall diversity [12]. Extracellular virus particles and inclusion bodies formed by SARS-CoV-2 components were identified in ultrathin sections of airway epithelial cells from patients with COVID-19 [6]. Notably, sequence alignments revealed that SARS-CoV-2 is closely related (86.9% identity) to the bat-derived SARS-like coronavirus bat-SL-CoVZC45, which was collected in China in 2003, while it is more distantly related to the first emergent SARS-CoV and Middle East Respiratory Syndrome Coronavirus (MERS-CoV).

### Host range of SARS-CoV-2

Most scientists view bats as the most likely reservoir of SARS-CoV-2 [13]. Studies comparing the total genome sequences indicate that SARS-CoV-2 may have evolved from a *Betacoronavirus* species found in Chinese horseshoe bats (*Rhinolophus sinicus*), since it shares up to 96.2% identity with some strains detected in bats [4]. Accordingly, bats are presumed to be the natural reservoir from which SARS-CoV-2 originated and spread to humans via an intermediary host [14]. However, researchers have not determined which animal is responsible for the final transmission to humans. Bats are the natural hosts for some of the most dangerous viruses, including Ebola, Marburg, rabies, Hendra, and Nipah [15]. Due to their special immunological features, bats are naturally infected but do not exhibit clinical signs of these diseases [16].

### SARS-CoV-2 transmission routes

SARS-CoV-2 is highly infectious and has become a threat worldwide. SARS-CoV-2 is mainly transmitted by direct contact and respiratory droplets [17–19]. However, it can also be transmitted via the fecal–oral route, mother-to-child route and aerosol route [20, 21]. Moreover, if people who are susceptible to disease touch freshly contaminated fomites on surfaces such as door handles and phones and then touch their own oral mucosa, nasal cavity or conjunctiva, indirect contact transmission might occur. Transmission via the aerosol route mainly occurs in confined and nonventilated spaces.

### Structure of SARS-CoV-2

**Fig. 1:**
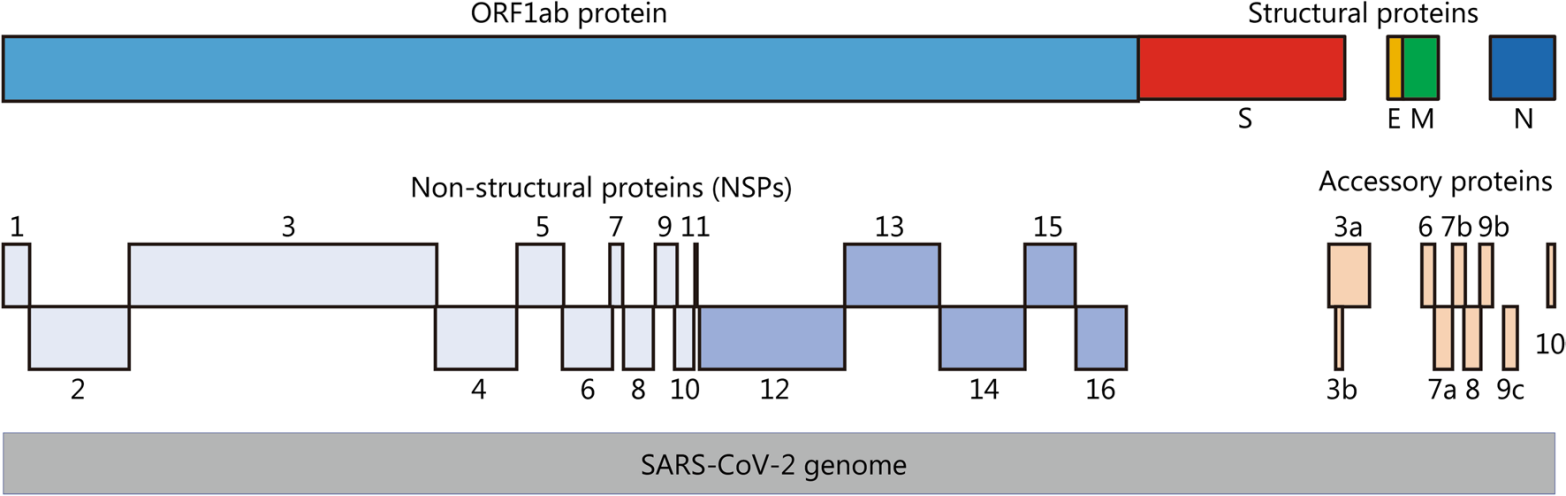
Organization of the SARS-CoV-2 genome. S spike protein; E envelope protein; M membrane protein; N nucleocapsid protein

**Fig. 2:**
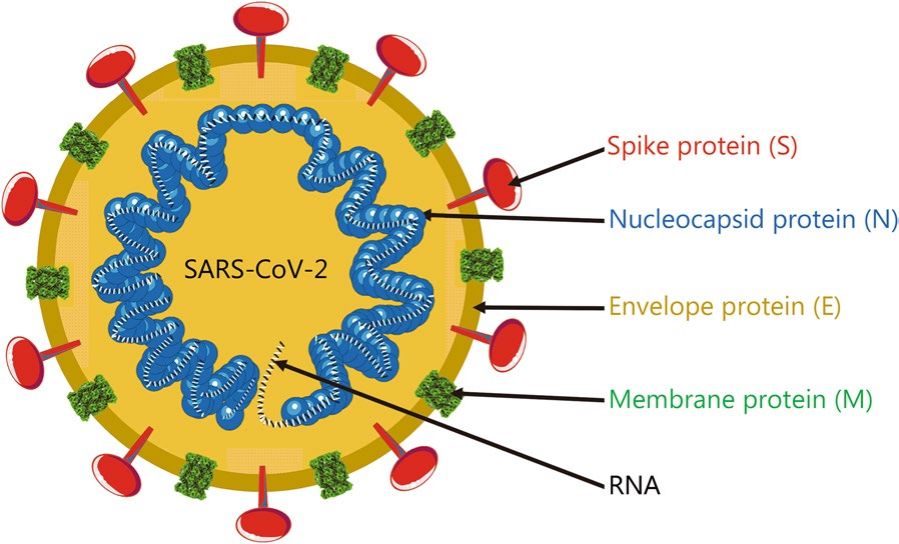
Structure of SARS-CoV-2

The SARS-CoV-2 genome is composed of a single positive-strand RNA, which is replicated with the assistance of nonstructural proteins (NSPs) [22]. As an RNA virus, SARS-CoV-2 requires an RNA polymerase to replicate its genome, and this polymerase lacks a proofreading function. As a result, the inherent mutation rate during replication in the host or intermediate host is high. Additionally, the high gene recombination rate between different coronaviruses is one of the reasons for their high diversity [23]. As shown in Fig. 1, the SARS-CoV-2 genome encompasses 29,903 nt, with multiple genes that encode 29 proteins. The coding sequence is flanked by two untranslated terminal regions (UTRs) and contains 14 annotated open reading frames (ORFs). The first two 5′-ORFs, ORF1ab and ORF1a, account for approximately two-thirds of the genome and encode 16 NSPs that are responsible for viral replication [23, 24]. The SARS-CoV-2 genome has a GC content of 38% [24]. The encoded sequences include the 5′ UTR, replicase complex (orf1ab), 4 structural proteins (spike protein, membrane protein, envelope protein and nucleocapsid protein), 8 accessory proteins [25], 3′ UTR and some unstructured open reading frames [3-chymotrypsin-like protease (3CLpro)], also called the main protease (Mpro), papain-like protease (PLpro), helicase and RNA-dependent RNA polymerase (RdRp)). These proteins are presumed to play a role in virus replication and pathogenesis [26]. The SARS-CoV-2 virus particles have obvious spinous processes that are 9–12 nm long such that the viral particle resembles the sun’s corona, from which its name was derived. SARS-CoV-2 virions appear polymorphic and mostly spherical under an electron microscope. All virus particles have core–shell structures with a diameter ranging from 60 to 140 nm [6]. Similar to the original emergent SARS-CoV, the four structural proteins of SARS-CoV-2 play important roles in viral infection (Fig. 2). These proteins may help us develop prophylactic vaccines that prevent viral cell entry, which is very important.

The spike protein is one of the 4 structural proteins that play important roles in forming the exterior of SARS-CoV-2 particles and protecting the internal RNA. The spike protein is a typical type I transmembrane glycoprotein constituting a unique spike-like structure on the surface of the virus. Spike protein is composed of S1 and S2 subunits [27]. S1 constitutes the globular head of the spike protein, providing the N-terminal domain (NTD) and the receptor-binding domain (RBD), which is responsible for recognizing the host cell receptor [28]. S1 is crucial for determining the spike orientation and host range. S2 forms the stem of the spike protein and participates in membrane fusion. The S2 subunit contains 3 functional groups, including a fusion peptide (FP) and a peptide repeat sequence (HR1 and HR2). After the RBD located in the tip of S1 binds to the receptor, the FP in S2 is inserted into the host cell membrane and changes the conformation, inducing the formation of a six-helix bundle (6HB) by HR1 and HR2, resulting in the fusion of the viral membrane with the cell membrane. The spike protein [29] forms obvious spikes on the surface of the virus. Some of the spikes extend and attach to angiotensin-converting enzyme 2 (ACE2), after which the virus invades the cell [30]. A special 12-base sequence (ccucggcgggca) in the spike protein gene has been identified that may help the spike protein bind more tightly to human cells. In vitro experiments also showed that if the host cell lacks the ACE2 protein, then it cannot be infected [31]. Several teams are currently researching and developing drugs to prevent this protein from attaching to human cells. Similar to SARS-CoV and MERS-CoV, the spike protein of SARS-CoV-2 and its RBD contained are still the main targets of vaccine development [32]. The structure of the SARS-CoV-2 spike protein has been solved and understood in some detail.

The membrane protein and E protein are structural proteins that form the outer shell of the virion. Additionally, the E protein binds other proteins inside the cell, further facilitating infection. The membrane protein is the most abundant structural protein of the virus, which determines the shape of the virion [33]. It has a larger C-terminal inner domain and a small N-terminal glycosylated extracellular domain. Moreover, the membrane protein is a dimer in the virion and adopts two different conformations to promote membrane bending and bind to the nucleocapsid together. A few transmembrane E proteins existed in the virion. E protein is composed of a C-terminal internal domain and an N-terminal extracellular, and possesses ion channel activity, which is helpful for the assembly and release of virions. Recombinant viruses lacking the E protein may not be viable.

In addition, nucleocapsid proteins are connected in the form of a long helix, wrapping the internal RNA. Their main function is to maintain the stability of the RNA inside the virion. Nucleocapsid protein is the only protein that forms the nucleocapsid. It consists of independent N- and C-terminal domains (NTD and CTD, respectively). These domains use different mechanisms to bind RNA in vitro, indicating that optimal RNA binding requires the participation of these two domains. The nucleocapsid protein also binds to the key components of the replicase complex, nsp3 and membrane protein, and these protein interactions may help package the protein-coated genome into viral particles [34]. Previous studies have also shown that the nucleocapsid protein plays a role in the invasion of SARS-CoV-2 and elicits an immune response.

### Pathogenic mechanism of SARS-CoV-2

The pathogenic mechanism of SARS-CoV-2 has not been fully clarified. The process of SARS-CoV-2 infection is the same as other coronaviruses. First, the spike protein binds to a specific receptor on the human cell membrane. Second, the virus enters the cell through receptor-mediated endocytosis and begins to replicate. Then, newly formed virions leave the host cell and infect other host cells [12]. Pre-studies have found that the receptor used by SARS-CoV-2 during invasion is the same as that of SARS-CoV, ACE2, suggesting that the host range and tissue tropism of the two viruses may be similar [29, 35]. Researchers have identified that the mechanism through which SARS-CoV-2 enters the cell is related to ACE2, the proprotein convertase furin, transmembrane serine protease 2 (TMPRSS2) and the lysosomal protease cathepsin. In the virion, the spike protein exists as a trimer; the S1 head is located at the top of the trimeric membrane-fused S2 stalk, which contains the RBD and specifically recognizes ACE2. Additionally, the virus uses the cell surface protease TMPRSS2 or the lysosomal protease cathepsin to dissociate S1 and activate the S1/S2 boundary through proteolysis. The mechanism SARS-CoV-2 uses to enter the cell reduces its dependence on the surface protease of the target cell. At the same time, pre-activation by furin and the high affinity of the RBD for ACE2 enable SARS-CoV-2 to effectively enter the cell while evading immune surveillance. These characteristics enable the rapid spread of the virus, aggravating the symptoms of patients and even causing death [36]. Some scientists have found that the spike protein of SARS-CoV-2 has a much higher affinity for binding to human ACE2 than that of SARS-CoV [29], which may be the main reason why SARS-CoV-2 is more infectious than SARS [37, 38]. Based on current research results, the interaction between the spike protein and ACE2 is an important premise for SARS-CoV-2 to invade cells [29]. At present, pathological research on the long-term existence of COVID-19 is mainly focused on the length of time the virus persists in the body and the long-term existence of viral RNA. An analysis of 26 patients revealed no critically ill patients, but SARS-CoV-2 and IgG antibodies coexisted for up to 50 days in two patients [39]. Additionally, the long-term existence of syncytia in infected cells and the continued existence of viral RNA in lung cells and endothelial cells may cause the virus to exist in the body for a long time [40].

### Variants of SARS-CoV-2

All viruses, including SARS-CoV-2, change over time. Most of the changes have little effect on virus properties (Table 1). However, some changes may affect the characteristics of the virus, such as the ease with which it spreads, the severity of the associated disease, and the efficacy of vaccines, therapeutic drugs, diagnostic tools or other public health and social measures. A number of variants have become variants of concern (VOC) or variants of interest (VOI), reclassifying VOI/VOC for scientists, health workers and the general public. VOC and VOI are the main focuses because they may significantly change the nature of the virus.

**Table 1:** SARS-CoV-2 variants (the data are from the WHO website, as of October 12, 2021)

WHO label	Pango lineages	Additional amino acid changes monitored^a^	Earliest documented samples	Date of designation
*Variants of Concern (VOC)*				
Alpha	B.1.1.7	+ S: 484K + S: 452R	United Kingdom, Sep-2020 [41, 42]	18-Dec-2020
Beta	B.1.351 B.1.351.2 B.1.351.3	+ S: L18F	South Africa, May-2020 [43–45]	18-Dec-2020
Gamma	P.1 P.1.1 P.1.2	+ S: 681H	Brazil, Nov-2020 [46]	11-Jan-2021
Delta	B.1.617.2 AY.1 AY.2	+ S: 417N	India, Oct-2020 [47]	VOI: 4-Apr-2021 VOC: 11-May-2021
*Variants of Interest (VOI)*				
Eta	B.1.525		Multiple countries, Dec-2020	17-Mar-2021
Iota	B.1.526		United States of America, Nov-2020	24-Mar-2021
Kappa	B.1.617.1		India, Oct-2020 [48]	4-Apr-2021
Lambda	C.37		Peru, Dec-2020	14-Jun-2021
Mu	B.1.621		Colombia, Jan-2021	30-Aug-2021
*Reclassifying VOIs/VOCs*				
	B.1.427 B.1.429^b^		United States of America, Mar-2020	VOI: 5-Mar-2021 Alert: 6-Jul-2021
	P.2^b^		Brazil, Apr-2020	VOI: 17-Mar-2021 Alert: 6-Jul-2021
	P.3^b^		Philippines, Jan-2021	VOI: 24-Mar-2021 Alert: 6-Jul-2021
	R.1 R.2		Multiple countries, Jan-2021	07-Apr-2021
	B.1.466.2		Indonesia, Nov-2020	28-Apr-2021
	B.1.621		Colombia, Jan-2021	26-May-2021
	AV.1		United Kingdom, Mar-2021	26-May-2021
	B.1.1.318		Multiple countries, Jan-2021	02-Jun-2021
	B.1.1.519		Multiple countries, Nov-2021	02-Jun-2021
	AT.1		Russian Federation, Jan-2021	09-Jun-2021
	C.36.3 C.36.3.1		Multiple countries, Jan-2021	16-Jun-2021
	B.1.214.2		Multiple countries, Nov-2020	30-Jun-2021
	B.1.1.523		Multiple countries, May-2020	14-Jul-2021
	B.1.620		Multiple countries, November 2020	14-Jul-2021
	C.1.2		South Africa, May 2021	01-Sep-2021
	B.1.617.1^b^		India, Oct-2020	VOI: 4-Apr-2021 VUM: 20-Sep-2021
	B.1.526^b^		United States of America, Nov-2020	VOI: 24-Mar-2021 VUM: 20-Sep-2021
	B.1.525^b^		Multiple countries, Dec-2020	VOI:17-Mar-2021 VUM: 20-Sep-2021
	B.1.630		Dominican Republic, Mar-2021	12-Oct-2021

^a^Significant monitored spike (S) amino acid changes have been reported in a small number of sequencing samples

^b^Former VOIs Epsilon (B.1.427/B.1.429), Zeta (P.2), Theta (P.3). VUM variants under monitoring

VOC have been shown through a comparative evaluation to be associated with one or more of the following changes of global public health significance: (1) to increase the prevalence of communicable or harmful changes in COVID-19 epidemiology; (2) increased toxicity or altered clinical manifestations; and (3) a decrease in the effectiveness of public health and social measures or existing diagnostics, vaccines and therapies.

VOI: (1) Predicted or known genetic changes that affect viral characteristics, such as transmissibility, disease severity, immune escape, and diagnostic or therapeutic escape; (2) identified causes of significant community transmission or clusters of COVID-19 cases, or other significant epidemiological effects, in multiple countries with increasing relative prevalence and increasing number of cases over time, suggest emerging risks to global public health.

Given the evolution of SARS-CoV-2 and our understanding of the effects of mutations, these working definitions may be adjusted periodically. Where necessary, variants that do not meet all the criteria listed in these definitions may be designated VOI/VOC, and those variants that have a reduced risk relative to other popular variants may be reclassified. Previously designated VOIs or VOCs, which have been conclusively shown to no longer pose a significantly increased risk to global public health compared to other prevalent SARS-CoV-2 variants, can be reclassified. The SARS-CoV-2 variant with genetic changes is suspected to affect viral characteristics, and some studies have indicated that it may pose a risk in the future, but evidence of phenotypic or epidemiological effects is currently unclear, requiring enhanced surveillance and repeated evaluation pending new evidence.

Since 2020, several variants of SARS-CoV-2 have appeared. Researchers have identified hundreds of mutations in the residues of the spike protein, but more mutation sites are located in the RBD. Chen et al. [49] conducted research on the six SARS-CoV-2 subtypes that appeared before; among them, clusters IV, V, and VI exhibited significantly greater infectivity. In addition, the author also predicted some residues (452, 489, 500, 501, and 505) that may be mutated in the future, and these residues have a high probability of producing more contagious SARS-CoV-2. Similarly, Daniloski et al. [50] studied the transduction rate of a SARS-CoV-2 variant (D614G); compared with wild-type SARS-CoV-2, D614G exhibited significantly increased transduction of A549^ACE2^ and Huh7.5^ACE2^ cells, indicating increased infectivity. Li et al. [51] studied the infectivity of 80 variants and 26 glycosylation modification sites, and the variant containing D614G and another amino acid was more infectious. Most of the variants caused by amino acid changes in the receptor binding region are less infectious, but some variants are resistant to partially neutralizing antibodies, such as A475V, L452R, V483A and F490L. In addition, the lack of glycosylation of N331 and N343 significantly reduces the infectivity of the virus. Among the four SARS-CoV-2 substrains discovered in the United States, studies have shown that Clusters A and D increase contagiosity, while Clusters B and C reduce contagiosity. Most importantly, when infected with SARS-CoV-2, the immune systems of women are more sensitive than those of men [52]. These results have reference value for the development of vaccines.

### Clinical presentations of COVID-19

Based on the current epidemiological data, the average incubation period of COVID-19 is 14 days. The most common symptoms at the onset of COVID-19 are fever, dry cough and fatigue. However, a few patients also present with nasal congestion, rhinorrhea, pharyngeal pain, myalgia or diarrhea. Asymptomatic infections have also been identified. In addition, patients admitted to the ICU were more likely to report dyspnea and/or hypoxemia, with some of them rapidly developing acute respiratory distress syndrome (ARDS), septic shock, metabolic acidosis, coagulopathy and multiple organ failure. Additionally, leukopenia and thrombocytopenia may occur in these severe cases [53]. Notably, severe cases are characterized by moderate to low fever or even no obvious fever. In contrast, mild cases are only characterized by a low fever, slight fatigue and usually no pneumonia [13]. Moreover, manifestations in some children and neonates may be atypical, including gastrointestinal symptoms such as vomiting and diarrhea, or only with mental weakness and shortness of breath.

Recently, relevant documents of the Chinese government clearly state that imaging results should be included as one of the criteria for a diagnosis of COVID-19. From the perspective of imaging, patients with COVID-19 of different severities have different presentations. Usually, no abnormal findings on chest CT are observed in patients with mild cases, but many patients show patchy ground-glass opacity, which is mainly focal and scattered bilaterally in the lungs. However, unilateral involvement is also observed. Lesions are commonly detected in the lower lobes, while the upper lobes may also be partially involved.

In severely and critically ill patients with COVID-19 pneumonia, bilateral multiple opacities of mixed density or ground-glass appearance with clear or ambiguous boundaries are common findings in chest CT images. The central and peripheral zones of the lungs are usually involved, with subpleural regions most commonly involved, and consolidation of different extents are observed within the opacities. Moderately ill patients have a much lower volume fraction of the lesions in the lungs than severely and critically ill patients, and severely ill patients have a lower value than critically ill patients. According to the evolution of imaging findings on chest CT, the disease is subclassified into four periods: early period, progression period, peak period and absorption period. Most patients with COVID-19 can be cured, but some patients with severe cases may develop acute respiratory distress syndrome, and many die [53]. Clinical studies have shown that deceased patients with COVID-19 exhibited typical pathological changes in the lung parenchyma that eventually led to progressive hypoxemia, lactic acidemia, ARDS and acute respiratory failure [54]. Autopsy of patients who died of COVID-19 revealed that the blood vessels of the human body exhibit some altered features after COVID-19, such as pulmonary vascular endotheliitis, vascular thrombosis, microvascular disease and alveolar capillary occlusion, which have caused harm to the human body [55]. COVID-19 is generally more severe in older or immunocompromised patients [13]. Additionally, women are less likely to develop severe COVID-19 than men [54].

## Concepts and methods of vaccine development

Prior to the successful development of a vaccine, no drugs specifically targeting SARS-CoV-2 were available in clinical practice, and the main measure to control the epidemic is still quarantine. At the same time, wearing masks, using liquid disinfectants and other measures effectively reduce the spread of SARS-CoV-2. The best approach to control epidemics is an effective vaccine. Teams all over the world are focusing on vaccine research and development during the epidemic. Fortunately, they have achieved some gratifying results. To date, some types of vaccines have been developed and put on the market, making them the most effective measures to combat the epidemic.

After confirming the epidemic situation, the Ministry of Science and Technology of the People’s Republic of China promoted research through several technical routes, including inactivated virus vaccines, recombinant protein vaccines, viral vector vaccines, and nucleic acid vaccines [56–58] (Table 2), to guarantee the success of vaccine research and development. At present, more than 400 teams worldwide have launched vaccine research based on these five technical routes. We have summarized the research and progress on these vaccines, hoping to identify safer and more effective new paradigms for vaccine development.

**Table 2:** Features of vaccines [59, 60]

Type	Principle	Advantages	Disadvantages
Viral vector vaccines	Insertion of the gene encoding the protective exogenous antigen into a viral vector to express the target protein in the body	Can insert long exogenous genes and use multiple inoculation routes High delivery efficiency Both cellular and mucosal immunity can be induced Easy to manufacture No adjuvant is required	The viral vector may interfere with the immune response to the target antigen Preexisting immunity may interfere with the vaccine effect Low safety
DNA vaccines	DNA vaccines are based on a eukaryotic expression vector encoding a certain protein antigen which is injected into the animal directly, so that the exogenous gene is expressed in vivo, and the antigen activates the body's immune system, thereby inducing specific humoral and cellular immune responses	Uses the host protein translation system to generate target antigens Induces both humoral and cellular immune responses Low cost Easy to mass manufacture and no need for cold chain transportation	There is a potential safety issue of DNA integration into the host genome
RNA vaccines	The mRNA vaccines use a synthetic mRNA encoding the translated antigen that is formulated in vitro and delivered into the body for translation into antigenic proteins by host cells	Easy and fast to produce Much safer than DNA vaccines Higher immunogenicity than DNA vaccines Can deliver multiple antigens at the same time	Poor stability May cause adverse reactions
Live-attenuated vaccines	A virus that is less virulent but still immunogenic and capable of replicating inside the body	Strong immunogenicity Sustained systemic and mucosal immune responses can be induced	Low safety Difficult to preserve and easy to inactivate Slow development and high screening effort The timing of the attenuation is unknown Large-scale culture of highly virulent pathogens must be done in a BSL3 facility
Inactivated virus vaccines	A whole-virus vaccine made from cultured wild-type viruses by physical or chemical inactivation processes	Easy to obtain Shorter cycle of early research Mature technology Similar to live viruses No concern surrounding reversion to virulence Much safer than live-attenuated vaccines	High risks BSL3 facility Can cause harmful reactions The immune effect is poor, requiring multiple doses and times Adjuvant may be required
Recombinant protein vaccines	It consists of purified recombinant proteins	Clear composition Excellent safety High stability Scalable production	Poor immunogenicity Adjuvants are indispensable
Bionic nanoparticle vaccines	It consists of purified recombinant proteins and bionic nanoparticles	Clear composition Excellent safety High stability Scalable production High efficiency	

**Fig. 3:**
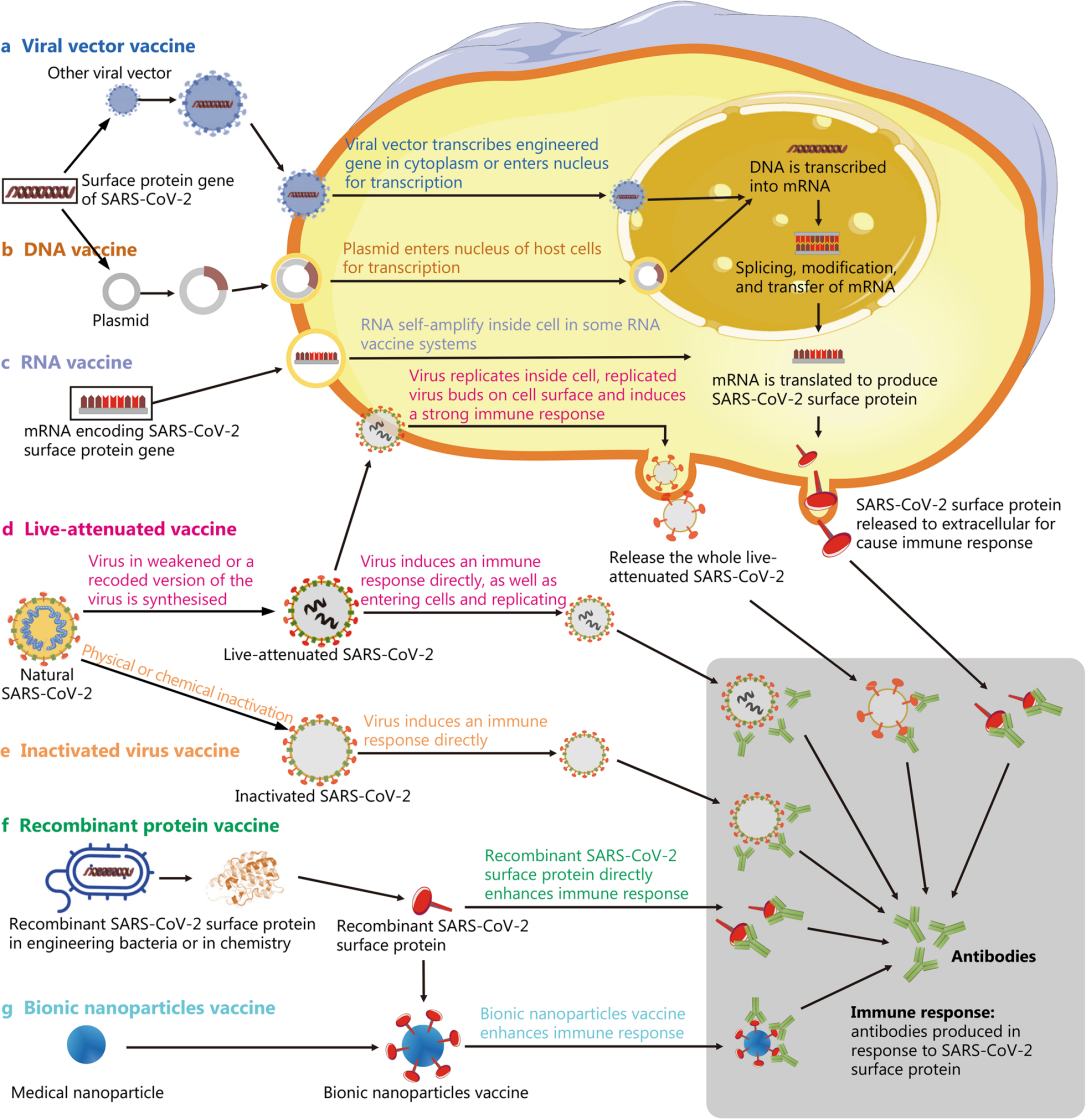
Schematic diagram showing the principles of various vaccines. **a** Viral vector vaccines are produced by integrating the SARS-CoV-2 antigenic gene fragment into viruses with very low pathogenicity. The gene is then transcribed in the cytoplasm or enters the nucleus for transcription, and finally, SARS-CoV-2 surface proteins are produced and cause an immune response. **b** DNA vaccines are produced using technology similar to a, but the vector is a plasmid. **c** mRNA vaccine is based on a synthetic mRNA encoding the SARS-CoV-2 antigen that is produced in vitro and delivered into the body. Then, it is translated into a protein antigen by cells and causes an immune response in the human body. **d** Live-attenuated vaccines are prepared by continuous passage to weaken the virulence of live viruses. The attenuated virus then directly induces an immune response by entering cells and replicating to induce the production of antibodies against SARS-CoV-2 surface proteins. **e** Inactivated virus vaccines are generated from the natural virus, which is inactivated using physical or chemical methods. The killed virus then directly induces an immune response. **f** Recombinant protein vaccines are based on injecting recombinant SARS-CoV-2 surface proteins directly into the living body to induce an immune response. **g** Based on **f**, bionic nanoparticle vaccines use nanoparticles composed of a biodegradable material to replace the nucleic acid and proteins of the viral core, while the outer shell contains recombinant viral surface proteins attached using synthetic biology. These two parts then form a virus-like structure through self-assembly and are injected into the body to induce an immune response

Because of the SARS-CoV-2 pandemic, vaccine development suddenly became a focus of global research. However, the time needed to develop a vaccine is very long. Years to more than a decade is often required from preclinical research to the final marketed vaccine (Fig. 3). The shortest development period before SARS-CoV-2 was that of the mumps vaccine, which took 5 years to market. In contrast, after several months of research and development of SARS-CoV-2 vaccines, several candidates have entered the clinic worldwide (Tables 3, 4, 5). This unprecedented speed also required governments to adopt a different approval process to ensure the safety, efficiency and controllable quality of these new vaccines. According to the WHO, vaccine development must undergo preclinical research, clinical application, clinical trial agency application, registered clinical trial, phase I clinical trial, phase II clinical trial, phase III clinical trial, vaccine marketing and vaccine production. The process is generally divided into five stages and 22 steps: (1) early design; (2) animal experiments; (3) Phase I clinical trial to understand the preliminary safety of the vaccine; (4) Phase II clinical trial to determine the immunization procedure and dose; and (5) Phase III clinical trials for more extensive vaccination trials and evaluation of side effects. More than 1000 volunteers are required in Phase III, and the shortest period is 3–5 months. Therefore, the rapid development of SARS-CoV-2 vaccines is a challenge. A change in research and development concepts and approval methods is also imperative to ensure that people are vaccinated as soon as possible.

**Table 3:** Advances in COVID-19 vaccine research (as of October 12, 2021)

Type	Institution and candidate	Product description	Trial phase	References
Viral vector vaccine	Janssen Pharmaceutical Companies/Beth Israel Deaconess Medical Center/Emergent BioSolutions/Catalent/Biological E/Grand River Aseptic Manufacturing (GRAM)/Sanofi/Merck	Ad26.COV2-S (or JNJ-78436725), Non replicating viral vector, Ad26 (alone or with MVA boost)	Authorized	[61]
	University of Oxford, Oxford Biomedica, Vaccines Manufacturing and Innovation Centre, Pall Life Sciences, Cobra Biologics, HalixBV, Advent s.r.l., Merck KGaA, the Serum Institute, Vaccitech, Catalent, CSL, and AstraZeneca/IQVIA	Non replicating viral vector; COVID-19 Vaccine AstraZeneca (formerly AZD1222), (formerly ChAdOx1), (Covishield in India)	Authorized	[62–67]
	CanSino Biologics/Beijing Institute of Biotechnology/Petrovax	Non-replicating viral vector; Adenovirus Type 5 vector (Ad5-nCoV), (Convidecia™)	Phase III	[68, 69]
	Gamaleya Research Institute	Adeno-based; (Gam-COVID-Vac) (Sputnik V)	Phase III	[70, 71]
	Shenzhen Geno-Immune Medical Institute	LV-SMENP-DC vaccine and antigen-specific CTLs	Phase I/II	*
	The University of Hong Kong/Xiamen University/Wantai Biological Pharmacy	Replicating viral vector, intranasal flu-based RBD (DelNS1-2019-nCoV-RBD-OPT1)	Phase II	*
	Cellid Co., Ltd./IAVI	AdCLD-CoV19	Phase I/II	*
	Israel Institute for Biological Research/Weizmann Institute of Science	rVSV-SARS-CoV-2-S vaccine	Phase I/II	*
	Aivita Biomedical, Inc./National Institute of Health Research and Development, Ministry of Health Republic of Indonesia	Dendritic cell vaccine AV-COVID-19	Phase I	*
	Shenzhen Geno-Immune Medical Institute	Covid-19/aAPC vaccine	Phase I	*
	City of Hope Medical Center	MVA-based SARS-CoV-2 vaccine; (COH04S1)	Phase I	*
	German Center for Infection Research (DZIF)/IDT Biologika GmbH/Universitätsklinikum Hamburg-Eppendorf/Philipps University Marburg Medical Center/Ludwig-Maximilians University of Munich	Non-replicating viral vector; MVA-S encoded	Phase I	*
	ImmunityBio/NantKwest	Non-replicating viral vector; [E1-, E2b-, E3-] hAd5-COVID19-Spike/Nucleocapsid	Phase I	*
	ReiThera/Leukocare/Univercells/National Institute of Infectious diseases Lazzaro Spallanzani	Non-replicating viral vector; replication defective simian adenovirus encoding SARS-CoV-2 S (GRAd-CoV2)	Phase I	*
	Vaxart/Emergent BioSolutions	Non-replicating viral vector; Oral Vaccine platform	Phase I	*
DNA vaccine	Zydus Cadila Healthcare Limited	DNA; (ZyCoV-D) plasmid vaccine	Phase III	*
	Osaka University/AnGes/Takara Bio/Cytiva/Brickell Biotech	DNA; (AG0301 & AG0302) plasmid vaccine + adjuvant	Phase II/III	*
	Inovio Pharmaceuticals/Beijing Advaccine Biotechnology,/VGXI Inc./Richter-Helm BioLogics/Ology Bioservices/International Vaccine Institute/Seoul National University Hospital/Thermo Fisher Scientific/Kaneka Eurogentec	DNA; (INO-4800) plasmid vaccine with electroporation	Phase II/III	*
	GeneOne Life Science	DNA; (GLS-5310)	Phase I/II	*
	Genexine Consortium (GenNBio, International Vaccine Institute, Korea Advanced Institute of Science and Technology (KAIST), Pohang University of Science and Technology (POSTECH)/Binex/PT Kalbe Pharma	DNA; (formerly GX-19) (GX-19N)	Phase I/II	*
	OncoSec Medical Incorporated/Providence Cancer Institute	(CORVax12), IL-12 expression platform + “S” glycoprotein	Phase I	*
	Symvivo	DNA; bacTRL-Spike	Phase I	*
RNA-based vaccine	BioNTech/Pfizer/Fosun Pharma/Rentschler Biopharma	3 LNP-mRNAs; BNT162	Authorized	[72–82]
	Moderna/National Institute of Allergy and Infectious Diseases (NIAID)/Biomedical Advanced Research and Development Authority (BARDA)/Lonza/Catalent/Rovi/Medidata/BIOQUAL/Baxter BioPharma Solutions	RNA; LNP-encapsulated mRNA (mRNA 1273), (TAK-919)	Authorized	[83–88]
	CureVac/Bayer/Novartis	RNA; mRNA (CVnCoV);	Phase III	*
	Arcturus/Duke-NUS/Catalent	RNA; mRNA; (LUNAR-COV19)	Phase I/II	*
	Imperial College London/VacEquity Global Health	RNA	Phase I/II	*
	People's Liberation Army (PLA) Academy of Military Sciences/Walvax Biotech	LNP-nCoVsaRNA; mRNA (ARCoV)	Phase I	*
	Providence Therapeutics Holdings Inc	PTX-COVID19-B vaccine	Phase I	*
Live-attenuated vaccine	Codagenix/Serum Institute of India	Single-dose, intranasal, live attenuated vaccine, (COVI-VAC)	Phase I	*
	Indian Immunologicals Ltd/Griffith University	Codon deoptimized live attenuated virus	Pre-clinical	*
	Meissa Vaccines	MV-014-210	Pre-clinical	*
	Mehmet Ali Aydinlar University/Acıbadem Labmed Health Services A.S.	Codon deoptimized live attenuated vaccines	Pre-clinical	*
Inactivated virus vaccine	Beijing Institute of Biological Products/Sinopharm	Inactivated, (BBIBP-CorV); Inactivated	Phase III	*
	Bharat Biotech/Indian Council of Medical Research/National Institute of Virology/Ocugen/Precisa Medicamentos	Whole-virion (COVAXIN) (BBV152)	Phase III	[89–91]
	Institute of Medical Biology, Chinese Academy of Medical Sciences	Inactivated	Phase III	[92]
	Research Institute for Biological Safety Problems, Republic of Kazakhstan	Inactivated, (QazCovid-in®)	Phase III	*
	Sinovac/Instituto Butantan/Bio Farma	Inactivated (inactivated + alum); CoronaVac (formerly PiCoVacc)	Phase III	[93, 94]
	Wuhan Institute of Biological Products/Sinopharm	Inactivated	Phase III	[95]
	Erciyes University	Inactivated; (ERUCOV-VAC)	Phase II	*
	Valneva/Dynavax/National Institute for Health Research, United Kingdom	Inactivated (Inactivated + CpG 1018), VLA2001	Phase I/II	*
	Shifa Pharmed Industrial Co	COVID-19 inactivated vaccine (COVIran Barekat)	Phase I	*
	Shenzhen Kangtai Biological Products Co.,Ltd./Beijing Minhai Biotechnology Co., Ltd	Inactivated SARS-CoV-2 vaccine (Vero cell)	Phase II	*
Recombinant protein vaccine	Anhui Zhifei Longcom Biopharmaceutical/Institute of Microbiology, Chinese Academy of Sciences	Adjuvanted recombinant protein (RBD-Dimer); (ZF2001)	Phase III	[96]
	Instituto Finlay de Vacunas	rRBD produced in CHO-cell chemically conjugate to tetanus toxoid	Phase III	*
	Medigen Vaccine Biologics Corp/NIAID/Dynavax	(FINLAY-FR-2) (SOBERANA 02); MVC-COV1901 vaccine injection; S-2 P protein + CpG 1018	Phase II	*
	Sanofi Pasteur/GSK	Protein subunit; spike protein, baculovirus production	Phase II	[97]
	West China Hospital, Sichuan University	RBD (baculovirus production expressed in Sf9 cells)	Phase II	*
	Biological E Ltd/Dynavax/Baylor College of Medicine	Protein subunit; (BECOV2)	Phase I/II	*
	Center for Genetic Engineering and Biotechnology (CIGB), Havana	CIGB-669 (RBD-AgnHB)	Phase I/II	*
	Federal Budgetary Research Institution (FBRI) State Research Center of Virology and Biotechnology "VECTOR"	Peptide vaccine, EpiVacCorona	Phase I/II	*
	Instituto Finlay de Vacunas	RBD + Adjuvant (FINLAY-FR-1) (SOBERANA 01)	Phase I/II	*
	Nanogen Pharmaceutical Biotechnology	Recombinant SARS-CoV-2 spike protein, aluminum adjuvanted	Phase I/II	*
	Shionogi & Co., Ltd./National Institute of Infectious Disease, Japan	Recombinant protein vaccine S-268019, baculovirus expression	Phase I/II	*
	VIDO-InterVac, University of Saskatchewan	Protein subunit, adjuvanted microsphere peptide, (COVAC-1 & COVAC-2)	Phase I/II	*
	Adimmune Corporation	Baculovirus-insect cells expression system, spike (S) protein (tAdimrSC-2f)	Phase I	*
	Center for Genetic Engineering and Biotechnology (CIGB), Havana	CIGB-66 (RBD + aluminum hydroxide)	Phase I	*
	Clover Biopharmaceuticals Inc./Dynavax	Protein subunit, native like trimeric subunit spike protein; (SCB-2019)	Phase I	[98, 99]
	Covaxx/University of Nebraska Medical Center (UNMC)/DASA/United Biomedical Inc. Asia	S1-RBD-protein; Multitope Peptide-Based Vaccine (MVP); UB-612	Phase I	*
	Razi Vaccine and Serum Research Institute	SARS-CoV-2 recombinant Spike protein vaccine (Razi Cov Pars)	Phase I	*
	University Hospital Tuebingen	SARS-CoV-2 HLA-DR peptides, (CoVAC-1)	Phase I	*
	Vaxine Pty Ltd/Flinders University/Oracle/Medytox/Sypharma/Oxford Expression Technologies	Protein subunit; recombinant spike protein with Advax adjuvant (COVAX-19)	Phase I	*
Bionic nanoparticles vaccine	Novavax/Emergent Biosolutions/Praha Vaccines/Biofabri/Fujifilm Diosynth Biotechnologies/FDB/Serum Institute of India/SK bioscience/Takeda Pharmaceutical Company Limited/AGC Biologics/PolyPeptide Group/Endo	Protein subunit; Full length recombinant SARs COV-2 glycoprotein nanoparticle vaccine adjuvanted with Matrix M	Phase III	[100, 101]
	Institute of Human Virology, Key Laboratory of Tropical Disease Control of Ministry of Education	Covalently conjugating the self-assembled 24-mer ferritin to the receptor binding domain (RBD) and/or heptad repeat (HR) subunits of the Severe Acute Respiratory Syndrome Coronavirus-2 (SARS-CoV-2) spike (S) protein	Pre-clinical or early research	[102]
	Novavax, Inc., 21 Firstfield Road, Gaithersburg	Constructed from the full–length, wild-type SARS–CoV-2 spike glycoprotein optimized for the baculovirus-*Spodoptera frugiperda* (Sf9) insect cell expression system	Pre-clinical or early research	[103]
	Department of Biochemistry, University of Washington, Seattle	SARS-CoV-2 RBD-I53-50 Nanoparticle	Pre-clinical or early research	[104]

^*^Indicates link of COVID-19 Treatment and Vaccine Tracker: https://covid-19tracker.milkeninstitute.org. For more details, please see: https://airtable.com/shrSAi6t5WFwqo3GM/tblEzPQS5fnc0FHYR/viwDBH7b6FjmIBX5x?blocks=bipZFzhJ7wHPv7x9z

**Table 4:** Vaccines already approved for the market (as of October 12, 2021)

Type	Institution and candidate	Product name	References	Status
mRNA-based vaccine	BioNTech/Pfizer	Comirnaty (also known as tozinameran or BNT162b2)	[105, 106]	Approved for use in: Bahrain, Brazil, New Zealand, Saudi Arabia, Switzerland
mRNA-based vaccine	Moderna/National Institutes of Health	mRNA-1273	[107–110]	Approved for use in: Switzerland
Recombinant adenovirus vector vaccine	Gamaleya Research Institute	Sputnik V (also known as Gam-Covid-Vac)	[111, 112]	Emergency use in: Albania, Algeria, Angola, Antigua and Barbuda, Argentina, Armenia, Azerbaijan, Bahrain, Bangladesh, Belarus, Bolivia, Bosnian Serb Republic, Cameroon, Congo Republic, Djibouti, Ecuador NEW, Egypt, Honduras, Gabon, Ghana, Guatemala, Guinea, Guyana, Hungary, India, Iran, Iraq, Jordan, Kazakhstan, Kenya, Kyrgyzstan, Laos, Lebanon, Maldives NEW, Mali, Mauritius, Mexico, Moldova, Mongolia, Montenegro, Morocco, Myanmar, Namibia, Nepal, Nicaragua (including Sputnik Light), North Macedonia, Pakistan, Palestinian Authority, Panama, Paraguay, Philippines, Russia (including Sputnik Light), San Marino, Serbia, Seychelles, Slovakia, Sri Lanka, St. Vincent and the Grenadines, Syria, Tunisia, Turkey, Turkmenistan, United Arab Emirates, Uzbekistan, Venezuela (including Sputnik Light) NEW, Vietnam, Zimbabwe
Non-replicating viral vector	Oxford University/AstraZeneca	Vaxzevria (also known as AZD1222, or Covishield in India)	[63, 113–118]	Approved for use in: Brazil
Recombinant adenovirus vector vaccine	CanSino Biologics/Academy of Military Medical Sciences	Convidecia (also known as Ad5-nCoV)	[119, 120]	Approved for use in: China
Non-replicating viral vector	Johnson & Johnson’s/Beth Israel Deaconess Medical Center	Ad26.COV2.S	[121–126]	Emergency use in: Bahrain, Brazil, Canada, Colombia, European Union, Greenland, Iceland, Liechtenstein, Moldova NEW, Norway, Philippines, South Africa, South Korea, Switzerland, Thailand, United States, Zambia
Synthetic peptide vaccine	Vector Institute	EpiVacCorona	[127]	Approved for use in: Turkmenistan
Protein subunit	Anhui Zhifei Longcom and the Institute of Medical Biology at the Chinese Academy of Medical Sciences	ZF2001	[128, 129]	Emergency use in: China, Uzbekistan
Inactivated virus	Beijing Institute of Biological Products	BBIBP-CorV	[130–132]	Approved for use in: Bahrain, China, United Arab Emirates
Non-replicating viral vector	Sinovac Biotech	CoronaVac (formerly PiCoVacc)	[128, 133]	Approved for use in: China
Non-replicating viral vector	Indian Council of Medical Research and the National Institute of Virology/Bharat Biotech	Covaxin (also known as BBV152 A, B, C)	[134]	Emergency use in: Botswana, Guatemala, Guyana, India, Iran, Mauritius, Mexico, Nepal, Nicaragua, Paraguay, Philippines, Zimbabwe
Inactivated virus	Wuhan Institute of Biological Products/Sinopharm	SARS-CoV-2 Vaccine (Vero cell), inactivated	[135]	Approved for use in: China
inactivated coronavirus vaccine	Chumakov Center at the Russian Academy of Sciences	CoviVac	[127]	Early use in: Russia
	Shenzhen Kangtai Biological Products	ChAdOx1	[136–139]	Emergency use in: China

**Table 5:** Vaccines entering Phase III/IV clinical trials (as of October 12, 2021)

Vaccine platform description	Type of candidate vaccine	Developers	Phase	References
Inactivated virus	CoronaVac; inactivated SARS-CoV-2 vaccine (Vero cell)	Sinovac Research and Development Co., Ltd	Phase IV	[140–145]
Inactivated virus	Inactivated SARS-CoV-2 vaccine (Vero cell)	Sinopharm + China National Biotec Group Co + Wuhan Institute of Biological Products	Phase III	[146, 147]
Inactivated virus	Inactivated SARS-CoV-2 vaccine (Vero cell), vaccine name BBIBP-CorV	Sinopharm + China National Biotec Group Co + Beijing Institute of Biological Products	Phase IV	[148]
Viral vector (Non-replicating)	ChAdOx1-S - (AZD1222) Covishield Vaxzevria	AstraZeneca + University of Oxford	Phase IV	[149]
Viral vector (Non-replicating)	Recombinant novel coronavirus vaccine (Adenovirus type 5 vector)	CanSino Biological Inc./Beijing Institute of Biotechnology	Phase IV	[150]
Viral vector (Non-replicating)	Gam-COVID-Vac Adeno-based (rAd26-S + rAd5-S)	Gamaleya Research Institute; Health Ministry of the Russian Federation	Phase III	
Viral vector (Non-replicating)	Ad26.COV2.S	Janssen Pharmaceutical Johnson & Johnson	Phase IV	[151]
Protein subunit	SARS-CoV-2 rS/Matrix M1-Adjuvant (Full length recombinant SARS CoV-2 glycoprotein nanoparticle vaccine adjuvanted with Matrix M) NVX-CoV2373	Novavax	Phase III	[152]
RNA based vaccine	mRNA-1273	Moderna + National Institute of Allergy and Infectious Diseases (NIAID)	Phase IV	[153]
RNA based vaccine	BNT162b2 (3 LNP-mRNAs), also known as "Comirnaty"	Pfizer/BioNTech + Fosun Pharma	Phase IV	[154]
Protein subunit	Recombinant SARS-CoV-2 vaccine (CHO Cell)	Anhui Zhifei Longcom Biopharmaceutical + Institute of Microbiology, Chinese Academy of Sciences	Phase III	
RNA based vaccine	CVnCoV vaccine	CureVac AG	Phase III	[155]
Inactivated virus	SARS-CoV-2 vaccine (Vero cell)	Institute of Medical Biology + Chinese Academy of Medical Sciences	Phase III	[156]
Inactivated virus	QazCovid-in® - COVID-19 inactivated vaccine	Research Institute for Biological Safety Problems, Rep of Kazakhstan	Phase III	*1
DNA based vaccine	nCov vaccine	Zydus Cadila	Phase III	*2
Inactivated virus	Whole-virion inactivated SARS-CoV-2 vaccine (BBV152); Covaxin	Bharat Biotech International Limited	Phase III	
Protein subunit	VAT00002: SARS-CoV-2 spike protein with adjuvant	Sanofi Pasteur + GSK	Phase III	[157]
Inactivated virus	Inactivated SARS-CoV-2 vaccine (Vero cell)	Shenzhen Kangtai Biological Products Co., Ltd	Phase III	*3
Protein subunit	FINLAY-FR-2 anti-SARS-CoV-2 vaccine (RBD chemically conjugated to tetanus toxoid plus adjuvant)	Instituto Finlay de Vacunas	Phase III	*4
Protein subunit	EpiVacCorona (EpiVacCorona vaccine based on peptide antigens for the prevention of COVID-19)	Federal Budgetary Research Institution State Research Center of Virology and Biotechnology "Vector"	Phase III	[158]
Protein subunit	RBD (baculovirus production expressed in Sf9 cells) Recombinant SARS-CoV-2 vaccine (Sf9 Cell)	West China Hospital + Sichuan University	Phase III	*5
RNA based vaccine	SARS-CoV-2 mRNA vaccine (ARCoV)	Academy of Military Science (AMS), Walvax Biotechnology and Suzhou Abogen Biosciences	Phase III	[159]
Protein subunit	CIGB-66 (RBD + aluminium hydroxide)	Center for Genetic Engineering and Biotechnology (CIGB)	Phase III	*6
Inactivated Virus	VLA2001	Valneva, National Institute for Health Research, United Kingdom	Phase III	*7
Protein subunit	Recombinant Sars-CoV-2 Spike protein, Aluminum adjuvanted (Nanocovax)	Nanogen Pharmaceutical Biotechnology	Phase III	[160]
Inactivated Virus	ERUCOV-VAC, inactivated virus	Erciyes University, Turkey	Phase III	*8
RNA based vaccine	mRNA-1273.351. A lipid nanoparticle (LNP)-encapsulated mRNA-based vaccine that encodes for a full-length, prefusion stabilized spike protein of the SARS-CoV-2 B.1.351 variant	Moderna + National Institute of Allergy and Infectious Diseases (NIAID)	Phase IV	[161]

^*^1: https://clinicaltrials.gov/ct2/show/NCT04530357

^*^2: http://www.ctri.nic.in/Clinicaltrials/pmaindet2.php?trialid=49858

^*^3: https://clinicaltrials.gov/ct2/show/NCT04852705

^*^4: https://search.bvsalud.org/global-literature-on-novel-coronavirus-2019-ncov/resource/en/ictrp-RPCEC00000347

^*^5: https://pactr.samrc.ac.za/TrialDisplay.aspx?TrialID=15727

^*^6: https://search.bvsalud.org/global-literature-on-novel-coronavirus-2019-ncov/resource/en/ictrp-RPCEC00000359

^*^7: https://search.bvsalud.org/global-literature-on-novel-coronavirus-2019-ncov/resource/en/ictrp-ISRCTN82411169

^*^8: https://clinicaltrials.gov/ct2/show/NCT04824391

### Viral vector vaccines

Vector-based vaccines are divided into replicative and nonreplicative vector-based vaccines. This type of vaccine is generated by integrating exogenous protective antigen-encoding genes into the genomes of viruses or bacteria whose harmful genes have been removed. A recombinant viral vector vaccine uses a virus as a carrier and effectively induces the organism to produce high-titer neutralizing antibodies. The mechanism involves the transcription of engineered genes in the nucleus and the production of SARS-CoV-2 surface proteins to elicit an immune response (Fig. 3a). Viral vectors commonly used as antigen delivery systems include DNA viruses such as vaccinia virus (VACV) [162–164], herpes simples virus (HSV) [165], and adenovirus [166–168], as well as RNA viruses such as influenza virus, vesicular stomatitis virus (VSV) [169–171] and yellow fever virus 17D (YF17D) [172].

Recombinant viral vector vaccines are generally live virus vaccines, and their vector functions as an adjuvant to induce humoral and cellular immunity at the same time. These vectors have become a research hotspot of novel genetically engineered vaccines because of their good safety, multiple inoculation routes and easy preparation. However, it is a relatively long process. Due to the infection of immune cells stimulates a humoral immune response, vaccines based on viral vectors produce a very strong cellular immune response because of their inherent adjuvant properties. By comparison with traditional vaccines (e.g., inactivated or attenuated virus), viral vector vaccines can be modified by specific targets to provide prolonged antigen presentation. The potential applications of viral vectors for humans ranged from infectious diseases to cancer treatments. Viral vectors also achieve high-levels of recombinant protein expression for the basis in modern vaccine development [157, 173, 174].

Currently, adenovirus vectors targeting COVID-19 mainly use first-generation Ad, which only provides short-term transgene expression in vivo. However, if prolongation of transgene expression is required without sacrificing the natural adjuvant properties of the virus, then enteric adenovirus vectors may be preferred because vector-mediated cells express only the vaccine antigen (spike) and not the Ad antigen. However, researchers have not clearly determined whether the pre-existing components of the vaccine are safe for humans. In addition, the recombinant adenovirus type-5 vector-based Ebola vaccine (AD5-EBOV) was approved by the National Medical Products Administration in 2017 [166, 175], while the recombinant vesicular stomatitis virus vector-based Ebola vaccine [ERVEBO (MSD)] was approved by the US Food and Drug Administration (FDA) in 2019. Moreover, recombinant viral vector vaccines against MERS-CoV [162, 176], influenza virus [163, 167], HIV [164, 168], Ebola [166, 177], Zika [170, 178] and Norwalk virus [171] are also undergoing active development.

Recombinant viral vector vaccines against COVID-19 are mostly generated by inserting the spike protein gene [37] and nucleocapsid protein gene of SARS-CoV-2 into the host virus genome to express the corresponding proteins in the body. Researchers have embedded the RBD of the spike protein into an influenza virus vector lacking pathogenic genes to construct a chimeric COVID-19 vaccine.

Currently, Chen and colleagues have emerged as the leader in the development of an adenovirus (Ad) type-5 vector-based vaccine against COVID-19. The results of the Phase I clinical trial of their vaccine showed that the Ad5 vector-based COVID-19 vaccine is tolerable and immunogenic in healthy adults. However, the vaccine still has deficiencies [68]. On the one hand, Ad5 may be rejected due to pre-existing immunity. Ad5 is a human-derived virus, and most people have been infected with Ad5 in their lives. Therefore, the anti-Ad5 antibodies that are generally present in humans may attack the Ad5 vector, thereby reducing the effectiveness of the vaccine [175]. On the other hand, the Ad5 vector vaccine cannot replicate in humans. As a result, the immunization dose of this vaccine must be increased to enhance its efficacy. However, high-dose immunization is not well tolerated. According to the official report of a Phase III trial conducted in September 2020, the interim analysis of a Phase III clinical trial of adenovirus vector type 5 (Ad5-nCoV) in Pakistan showed that the vaccine was 100% protective against severe COVID-19 after the administration of a single dose, with an overall protective efficacy of 74.8%.

In conclusion, pre-existing immunity should be considered when designing this type of vaccine. Viral vector vaccines also carry a certain biological risk. Additionally, the body's response to the carrier may interfere with the immune response to the target antigen. However, evidence for either problem has not been obtained in humans. Therefore, the identification of more effective antigens and targets, comprehensive use rare human adenovirus serotypes or adenoviruses from nonhuman primates, and combination with other types of vaccines for immunization will be more promising directions for the development of recombinant viral vector vaccines against COVID-19. On February 25, 2021, the recombinant novel coronavirus vaccine (Ad5-nCoV) Kevesa™ was approved by the State Medical Products Administration for conditional marketing in China. This vaccine is also the only vaccine that can be administered with a single injection program. The clinical Phase I/II data for this vaccine suggest that in addition to its high protective efficacy, a certain degree of side effects is also induced [150]. Research suggests that the AstraZeneca vaccine may cause an unusual reaction that causes clots to appear throughout the body, accompanied by low levels of platelets. However, vaccine regulators have argued that, in most settings, the benefits of the COVID-19 vaccines developed by AstraZeneca and Johnson & Johnson (J&J) far outweigh the small risk that they will cause an unusual and sometimes deadly clotting disorder. In addition, after being vaccinated with the Janssen COVID-19 vaccine, people aged 18–59 years are more likely to have reactogenicity symptoms than people aged more than 60 years. However, the symptoms are mostly mild to moderate and subside within 1–2 days. In addition, the probability of severe local or systemic reactogenicity symptoms (≥ grade 3) in vaccine recipients was 2.2%, which was higher than that in placebo recipients (0.7%). Early Phase I/II data from the Sputnik V COVID-19 vaccine were released in September 2020, showing that the immune response is induced at a level consistent with protection. A subsequent interim report of phase 3 data showed that 75% of the more than 20,000 participants were assigned to receive the vaccine, and approximately one-quarter of them had comorbidities.

### DNA vaccines

Nucleic acid vaccines are based on either DNA or mRNA. DNA vaccines are based on a recombinant eukaryotic expression vector encoding a certain protein antigen that is directly injected into animals such that the foreign gene is expressed in vivo, and the antigen activates the immune system, thereby inducing specific humoral and cellular immune responses (Fig. 3b). This approach delivers plasmids (e.g. pGX9501 in the case of INO-4800) containing the gene encoding the spike protein via intramuscular injection. An electrical pulse is employed to create transient pores in the cell membrane (namely electroporation) and then allowed these plasmids to enter the host cell smoothly. In cells, the plasmids begin to multiply, translate spike proteins, and activate the immune system of host. Inovio Pharmaceuticals has reported the results of a Phase I/II clinical trial of its INO-4800 vaccine [179], which is currently in Phase III trials. Notably, INO-4800 can be stored for more than 1 year at room temperature. Additionally, GLS-5310 (GeneOne Life Science, ClinicalTrials.gov Identifier: NCT04673149) and GX-19N (Genexine, ClinicalTrials.gov Identifier: NCT04715997) entered a Phase I clinical trial in Korea in 2021 [180]. Some DNA vaccines have been marketed, including animal flu vaccines and West Nile virus vaccines [181, 182].

Research on DNA vaccines began in the 1990s, when the most common route of administration was intramuscular (IM) or intradermal (ID) injections using conventional needles. Scientists have developed different DNA preparations, which are encapsulated in lipid nanoparticles containing cationic lipids and cholesterol. These DNA preparations are adsorbed onto polymers (such as polyethyleneimine) and adsorbed or encapsulated in biodegradable nanoparticles to increase the uptake of DNA molecules by cells [183]. Furthermore, "molecular adjuvants" have also been developed to enhance the prophylactic and treatment effects of DNA vaccines [184]. However, DNA vaccines also have many disadvantages. First, the DNA injected into the body is quickly degraded. Moreover, DNA vaccines pose a risk of autoimmunity, which has not been observed in nonprimates [185]. To date, DNA vaccines have not been approved for use in humans.

On May 6, 2020, The Innovation and Value Initiative (IVI), Inovio and the Korean National Institutes of Health (KNIH) announced a collaboration with the Coalition for Epidemic Preparedness Innovations (CEPI). They are testing the safety and immunogenicity of a DNA vaccine named INO-4800 in the first stage. Data released on May 20, 2020, suggested that the INO-4800 DNA vaccine was effective. According to Phase I data published in December 2020, INO-4800 exhibited excellent safety and tolerability and was immunogenic in 100% (38/38) of the vaccinated volunteers by eliciting humoral and/or cellular immune responses [186]. Phase II/III efficacy trials were scheduled to begin in July/August 2020 and are still subject to regulatory approval [187]. Moreover, an Indian company named Zydus Cadila announced that they had started a research project in cooperation with multiple teams in India and Europe on February 15, 2020, which aimed to develop a DNA vaccine against SARS-CoV-2. However, the potential safety problems of DNA vaccines cannot be ignored, mainly because the expression vector carrying the antigen-encoding gene can be integrated into the genome. DNA vaccines also have some advantages: no risk of infection [188], ease of development and production [188], long-term persistence of immunogens [189], and in vivo expression ensuring that proteins more closely resemble normal eukaryotic structures, with accompanying posttranslational modifications [189]. However, its disadvantages cannot be ignored, such as the potential for atypical processing of bacterial and parasite proteins [188] and potential to transfect nontarget cells, such as brain cells, when using nasal spray administration of plasmid DNA nanoparticles [190].

### RNA vaccines

In addition to delivering a DNA vector that must enter the nucleus to be transcribed, the mRNA encoding the target antigen can be synthesized in vitro and delivered into the body. In vivo, the mRNA is translated into antigen protein by cells and elicits both humoral and cellular immune responses in the human body (Fig. 3c). Over the past two decades, scientists have shown increased interest in the development of mRNA vaccines. Two main types of prophylactic mRNA vaccines have been developed: nonreplicating and self-amplifying mRNA vaccines. The nonreplicating mRNA vaccine contains 5′ and 3′ UTRs. Compared with the self-amplifying mRNA vaccine, the nonreplicating mRNA vaccine has the advantages of a simple structure, short RNA sequence and lack of requirement for additional proteins except for the antigen [191]. However, the injected naked mRNA may be degraded by ubiquitous extracellular ribonucleases. DNA vaccines must enter the nucleus to work, while mRNA vaccines only need to enter the cytoplasm to achieve the expression of the target antigen, and thus mRNA vaccines are theoretically safer than DNA vaccines. Moreover, mRNA is produced in vitro and does not need to be amplified in bacteria or cell culture; therefore, the process of producing mRNA vaccines is short and comparatively easy to monitor [192].

Based on the latest data, the National Institutes of Allergy and Infectious Diseases and Moderna Inc. (both from the USA) are in the leading position in mRNA vaccine research [193–196]. They are developing an mRNA vaccine named mRNA-1273. On March 27, 2020, the National Institutes of Health announced that Emory University in Atlanta began recruiting healthy adult volunteers aged 18 to 55 years to participate in a Phase I study of mRNA-1273 led by the National Institutes of Health [104]. On April 27, 2020, Moderna submitted an IND to the US FDA for a Phase I study of mRNA-1273. On May 12, 2020, Moderna received the FDA fast track certification for mRNA-1273. On May 18, 2020, Moderna announced favorable mid-term data from the Phase I trial of mRNA-1273, indicating that the vaccine is safe. On July 27, 2020, the mRNA-1273 vaccine entered Phase III clinical trials, and studies showed that the efficacy of the vaccine was 94.5%, indicating that the overall tolerance of the mRNA-1273 vaccine was good and that it had satisfactory safety and effectiveness [83].

In mid-May 2020, CureVac also announced that its candidate vaccine against SARS-CoV-2 produced high-level virus-neutralizing antibody titers after the administration of two preclinical 2 mg doses. In June 2020, CureVac started a Phase I/II clinical trial. On April 20, 2020, Arcturus Therapeutics and Duke-NUS Medical School conducted preclinical testing and then conducted the first human clinical trial. On April 27, 2020, those companies announced positive preclinical test data, which proved that the candidate mRNA vaccine LUNAR-COV19 had strong immunogenicity. On June 30, 2021, CureVac also announced results from the final analysis of its 40,000 subject international pivotal Phase IIb/III study (the HERALD study) of the first-generation COVID-19 vaccine candidate, CVnCoV. In the unprecedented context of 15 strains circulating within the study population at the time of final analysis, CVnCoV documented an overall vaccine efficacy of 48% (83 treated with the vaccine vs. 145 treated with the placebo) against COVID-19 disease of any severity, including single nonrespiratory mild symptoms. Significant protection was observed among participants in the age group of 18 to 60 years, with an efficacy of 53% (71 treated with the vaccine vs. 136 treated with the placebo) against disease of any severity and across all 15 identified strains; protection against moderate to severe disease was calculated to be 77% (9 treated with the vaccine vs. 36 treated with the placebo). In the same age group, CVnCoV provided 100% protection (0 treated with the vaccine vs. 6 treated with the placebo) against hospitalization or death. In participants aged greater than 60 years, who represented 9% of the analyzed participants, the available data did not enable a statistically significant determination of efficacy. In addition, several teams are also conducting relevant research (Table 3) [197]. BNT162b2 is a nucleoside-modified RNA vaccine. Studies have shown that the vaccine efficacy is 89–91% 15–28 days after the administration of the first dose of the BNT162b2 vaccine. In addition, the incidence of SARS-CoV-2 infection and symptomatic COVID-19 is significantly reduced in the early stages. In the case of vaccine shortages and scarce resources, a single dose of BNT162b2 vaccine may be administered to increase population coverage and reduce infection or morbidity rates [198, 199].

### Live-attenuated vaccines

Live-attenuated vaccines are based on originally pathogenic microorganisms that have been engineered for reduced virulence but still have the ability to replicate and elicit an immune response. The mechanism is based on a weakened or engineered version of the virus, which directly induces an immune response by entering cells and replicating, leading to the production of antibodies and cytotoxic T cells in response to SARS-CoV-2 proteins (Fig. 3d). This type of vaccine induces persistent systemic and mucosal immune responses due to its excellent immunogenicity. Existing live-attenuated vaccines include yellow fever vaccine, smallpox vaccine, measles vaccine, poliomyelitis vaccine, mumps vaccine, rubella vaccine, and varicella vaccine. In contrast with inactivated virus vaccines, which require at least one additional booster shot, live-attenuated vaccines only need to be administered once. They simulate the infection process of natural viruses and induce both humoral and cellular immunity, which exerts a stable and long-term protective effect on the body.

To date, four institutions are developing live-attenuated vaccines against COVID-19, including the Serum Institute of India, the largest vaccine company in the world. Live-attenuated vaccines take many years to develop, depending on the virus itself and the cells that are used to cultivate the attenuated strain. Generally, attenuated strains may appear when the cells are cultured to the 60th generation, and another 10–20 generations are usually needed to observe changes in the virus. In addition, very strict restrictions are in place for the culture of cells infected with live-attenuated vaccines. If cells are passaged too many times, the virus may cause certain changes in these cells. Finally, a subset of the viruses may develop atavistic mutations, reverting to pathogenicity. Previous studies have shown that live-attenuated vaccines against SARS revert to virulence after continuous passaging in cultured cells or mice [200].

As a result, live-attenuated vaccines pose a greater biosecurity risk. The application of a live-attenuated vaccine against COVID-19 is not recommended without sufficient evidence to ensure that the vaccine will not revert to virulence.

### Inactivated virus vaccines

An inactivated virus vaccine is prepared by culturing wild-type viruses or bacteria and then inactivating them physically or chemically. It may be composed of entire virions or bacterial cells or only their fragments. Inactivated virus vaccines that are currently used include the inactivated polio vaccine [201], inactivated Japanese encephalitis vaccine [202], inactivated hepatitis A vaccine [203], inactivated rabies vaccine [204], hand-foot-and-mouth disease vaccine [205], cholera vaccine [206], leptospirosis vaccine, bleeding heat vaccine, and forest encephalitis vaccines. For obvious reasons, inactivated vaccines are intrinsically much safer than live vaccines, and they generally have a more complete molecular spatial structure. However, the immunogenicity of inactivated virus vaccines is not as good as that of live vaccines, requiring increases in the dose and the number of inoculations to compensate.

Inactivated vaccines are generated from all bacteria or virions that are inactivated using physical or chemical methods, and this dead material directly induces an immune response (Fig. 3e). Therefore, the composition of the inactivated vaccine is relatively complex, including multiple immunogens that potentially cause adverse reactions in the inoculated person. However, when the antigen to choose is unclear, inactivated vaccines promote immunization possibilities. Similar to the first emergent SARS coronavirus, SARS-CoV-2 is a highly virulent infectious virus, and its inactivation process also must be performed in a laboratory with a biosafety level 3 or above. The high associated cost and risk also limit the development of inactivated vaccines. In addition, a series of problems have been noted, such as the staffing and financial means needed to develop vaccines, as well as the long development timeline. Moreover, human trials take a long time and are mired with unpredictable variables. By the time conventional vaccines enter clinical trials, the epidemic situation might be controlled or disappear. During this period, the virus will mutate frequently [207]. For SARS-CoV-2, if scientists solve the problems of effectiveness, durability, lack of cellular immunity and the short lifespan of its neutralizing antibody produced by inactivated vaccines, this type of vaccine is a feasible and stable development strategy. Results from CoronaVac trials show that a third dose of CoronaVac administered 6 or more months after a second dose effectively recalled a specific immune response to SARS-CoV-2, resulting in a remarkable increase in antibody levels and indicating that a two-dose schedule generates good immune memory. However, in the 3 μg group, neutralizing antibody titers induced by the first two doses decreased after 6–8 months to below the seropositive cutoff [208].

Inactivated virus vaccines have a long and successful history. They are the most immunogenic of the vaccine formulations. Moreover, inactivated vaccines are generally a safe, well-tolerated and effective treatment; however, this efficacy comes at a price in terms of potential safety issues. Based on available data, these vaccines may lead to immunopathology and adverse drug events, and the safety of vaccines must be carefully reviewed during animal studies and clinical trials [209]. The main disadvantages of inactivated vaccines are listed below. First, booster vaccines and adjuvants are often necessary when inactivated vaccines are administered [210]. For example, the dengue vaccine only contains dengue virus (DENV) structural proteins, hence fails to induce any immunity to nonstructural proteins. For optimal immunogenicity, the adjuvants are added to enhance reactogenicity. Multiple booster doses are required to provide long-term immunity, and they can be expensive to manufacture, as DENV does not grow to high titers in tissue culture cells. Above challenges make an inactivated DENV vaccine a less attractive vaccine candidate for use in DENV-endemic areas; however, they might be useful as travelers vaccine or as a part of a prime boost strategy with live or replicating vaccines [107, 108]. Second, inactivated virus vaccines may induce harmful immune and/or inflammatory responses. Currently, most of influenza vaccines are inactivated vaccines, which play an important role in protecting people from influenza virus infection. Inactivated vaccines against SARS-CoV have been prepared for in vivo experiments via some inactivated approaches, including formaldehyde, UV light, and β-propiolactone. He et al. [211] described that high-titer antibodies caused by inactivated SARS-CoV in immunized animals recognize the spike protein, especially the RBD in the S1 subunit, and potently block SARS-CoV entry. The safety of these inactivated vaccine for SARS-CoV have been evaluated, suggesting all vaccines can successfully induce serum neutralizing antibody production and significant reductions in the SARS-CoV titer after viral challenge. Even if few inactivated SARS-CoV-2 vaccines does not elicit a serious harmful immune response, it may enhance the infection of the mutated and/or another novel coronavirus through adverse drug events [212]. Finally, the immune response to other coronaviruses suggests that both cell-mediated and humoral immunity contribute to long-term protection. Inactivated vaccines usually induce weak cell-mediated immunity [213]. Compared to live attenuated vaccines, inactivated flu vaccines are more suitable for adults and the elderly [214].

Companies that are developing inactivated COVID-19 vaccines include CNBG in Beijing and Wuhan, Sinovac Biotech Co., Ltd., and the Institute of Medical Biology Chinese Academy of Medical Sciences. On February 22, 2020, Zhejiang Provincial Centers for Disease Control and Prevention, The First Hospital of Zhejiang Province, Hangzhou Medical Association and other teams working with enterprises selected the fourth-generation vaccine strain. On April 24, 2020, the inactivated COVID-19 vaccine developed by the Sinopharm China Wuhan Bioproducts Research Institute was the first inactivated COVID-19 vaccine to enter Phase II clinical trials worldwide. On April 27, 2020, the National Vaccine and Serum Institute was approved by the National Medical Products Administration to conduct combined clinical Phase I/II trials, and on April 29, the clinical Phase I healthy subject vaccination program was launched in Shangqiu City, Henan Province. On May 6, 2020, the inactivated vaccine BBIBP-CORV developed by Sinovac Biotech Co., Ltd., Key Laboratory of Comparative Medicine for Human Diseases, Ministry of Health, China and the Chinese Center for Disease Control and Prevention was proven to completely protect rhesus macaques against a lethal challenge with SARS-CoV-2 at a dose of 6 μg. At this time, BBIBP-CORV and other SARS-CoV-2 vaccine candidates were subsequently expected to begin Phase I, II, and III clinical trials [215]. On April 13, 2020, Sinovac Biotech received approval from governmental authorities to conduct both Phase I and Phase II human clinical trials of the BBIBP-CORV vaccine in China. On October 15, 2020, Phase I/II clinical data from the BBIBP-CORV inactivated vaccine were published in The Lancet, showing that the vaccine has satisfactory safety and tolerability [148]. Recently, Sinovac Biotech Co., Ltd. released preliminary data from a Phase III clinical study, which showed that the protective effect of a BBIBP-CORV inactivated vaccine exceeded 50% in Brazil and Turkey, with the highest protection reaching 91.25%. The BBIBP-CORV inactivated virus vaccine is administered to people aged 18 to 59 years and over 60 years, in whom researchers have found it to be safe and well tolerated [216]. The researchers also observed similar results in children and adolescents aged 3–17 years. The side effects and reactions from the vaccine were mild to moderate in severity and were temporary [140].

### Recombinant protein vaccines

Recombinant protein vaccines, also known as genetically engineered subunit vaccines, are generated by integrating the target genes of pathogenic microorganisms into a vector that is used to efficiently express antigen proteins in an unrelated industrial organism. The recombinant viral surface proteins are then injected directly into the body to induce an immune response (Fig. 3f). The antigenicity of these vaccines is closely related to their expression systems. Currently, the expression systems used to produce this type of vaccine mainly include bacteria, yeasts, insect cells and mammalian cells. This type of vaccine, such as the SARS-CoV Nucleocapsid protein subunit vaccine, is directly taken up by antigen-presenting cells with strong inherent adjuvant activity. Thus, they efficiently induce adaptive immune responses mediated by T and B cells [59]. Since the first recombinant vaccine produced in yeast was marketed in the 1980s, recombinant protein vaccines have become popular and have been developed rapidly. The most representative recombinant protein vaccines include hepatitis B virus vaccine [217], hepatitis E virus vaccine [218] and human papilloma virus vaccine [219]. In addition, recombinant protein vaccines against herpes zoster virus [220], foot-and-mouth disease virus, influenza virus and MERS coronavirus [221] are also under extensive development.

Recombinant protein vaccines have high intrinsic safety and excellent stability. Moreover, they can be produced on a very large scale, making them suitable for population-based vaccination campaigns. However, the shortcomings of recombinant protein vaccines, such as poor immunogenicity, limited immunization time, dependence on the time of immunization and adjuvant type, also result in challenges. At present, four main methods have been used to enhance the immunogenicity of recombinant protein vaccines: (1) agglomerating the vaccine in vitro and encapsulating it into liposomes or microspheres; (2) use of an adjuvant [222]; (3) fusing the virus epitope and immunoglobulin genes and expressing them as a single chimeric protein [223]; and (4) engineering the recombinant protein to self-assemble into a virus-like particle (VLP) [218].

At present, recombinant protein vaccines against COVID-19 are based on the spike protein from the surface of SARS-CoV-2 as the target antigen [37], which is expressed heterologously, purified, and formulated with an adjuvant. However, the nucleocapsid protein is also immunogenic and has been reported to be used for the development of recombinant protein vaccines against COVID-19. Several institutions are developing COVID-19 vaccines using this technical route (Table 3). Although recombinant protein vaccines are safe, some problems still exist. On the one hand, antibody-dependent enhancement (ADE) may develop and even aggravate the infection [224]. It is the risk of exacerbating COVID-19 severity via ADE, which is a potential hurdle for antibody-based vaccines and therapeutics. Because ADE can increase the severity of multiple viral infections, such as respiratory syncytial virus (RSV) [225, 226] and measles [227, 228]. Two distinct mechanisms of ADEs for viral infections. 1) ADEs can enhance antibody-mediated virus uptake into Fc gamma receptor IIa (FcγRIIa)-expressing phagocytic cells, leading to increased viral infection and replication; 2) excessive antibody Fc-mediated effector functions or immune complex formation that induced enhanced inflammation and immunopathology. ADEs has been generally observed in SARS, MERS and other human respiratory virus infections, such as RSV and measles, suggesting it is a real risk of ADEs as vaccines for SARS-CoV-2 [229]. However, clinical data have not yet fully established to explain ADEs in human pathology for COVID-19. Nevertheless, as an inevitable theoretical concern for COVID-19 vaccine development, this type of vaccine has attracted wide attention from researchers [230–234]. On the other hand, an appropriate adjuvant is essential. A study has suggested that MF59, AS03 and AF03 can not only induce balanced humoral and cellular immune responses but also induce a wide range of cross-reactions [89]. Thus, these three adjuvants may play roles in recombinant protein vaccines against COVID-19.

Cellular immunity plays a crucial role in clearing coronavirus infection. Notably, recombinant protein vaccines can induce humoral and mucosal immunity. The combined use of DNA vaccines and recombinant protein vaccines has been shown to effectively enhance the immunization efficacy [235]. Thus, the combined usage of recombinant protein vaccines and other COVID-19 vaccines may effectively stimulate systemic immune responses to SARS-CoV-2. Moreover, certain viral proteins expressed in bacteria with simple modifications naturally form multimeric subviral particles with good immunogenicity, suggesting that the development of COVID-19 vaccines through prokaryotic expression of subviral particle particles may become a research hotspot.

Nevertheless, highly efficient and safe recombinant protein vaccines against COVID-19 are difficult to obtain. If SARS-CoV-2 does not disappear quickly in the short term, recombinant protein vaccines may be used as a safer routine vaccine rather than an emergency vaccine. As of April 26, 2021, the novel coronavirus recombinant subunit protein vaccine was jointly developed by Gao Fu and colleagues at the Institute of Microbiology, Chinese Academy of Sciences and Anhui Zhifei Longkoma Biopharmaceutical Co., Ltd. The vaccine was generally well tolerated in adults and produced antibodies against the wild-type SARS-CoV-2 strain in vitro. However, the vaccine did not cause a strong neutralizing response to the virus in the elderly [236].

### Bionic nanoparticle vaccines

**Fig. 4:**
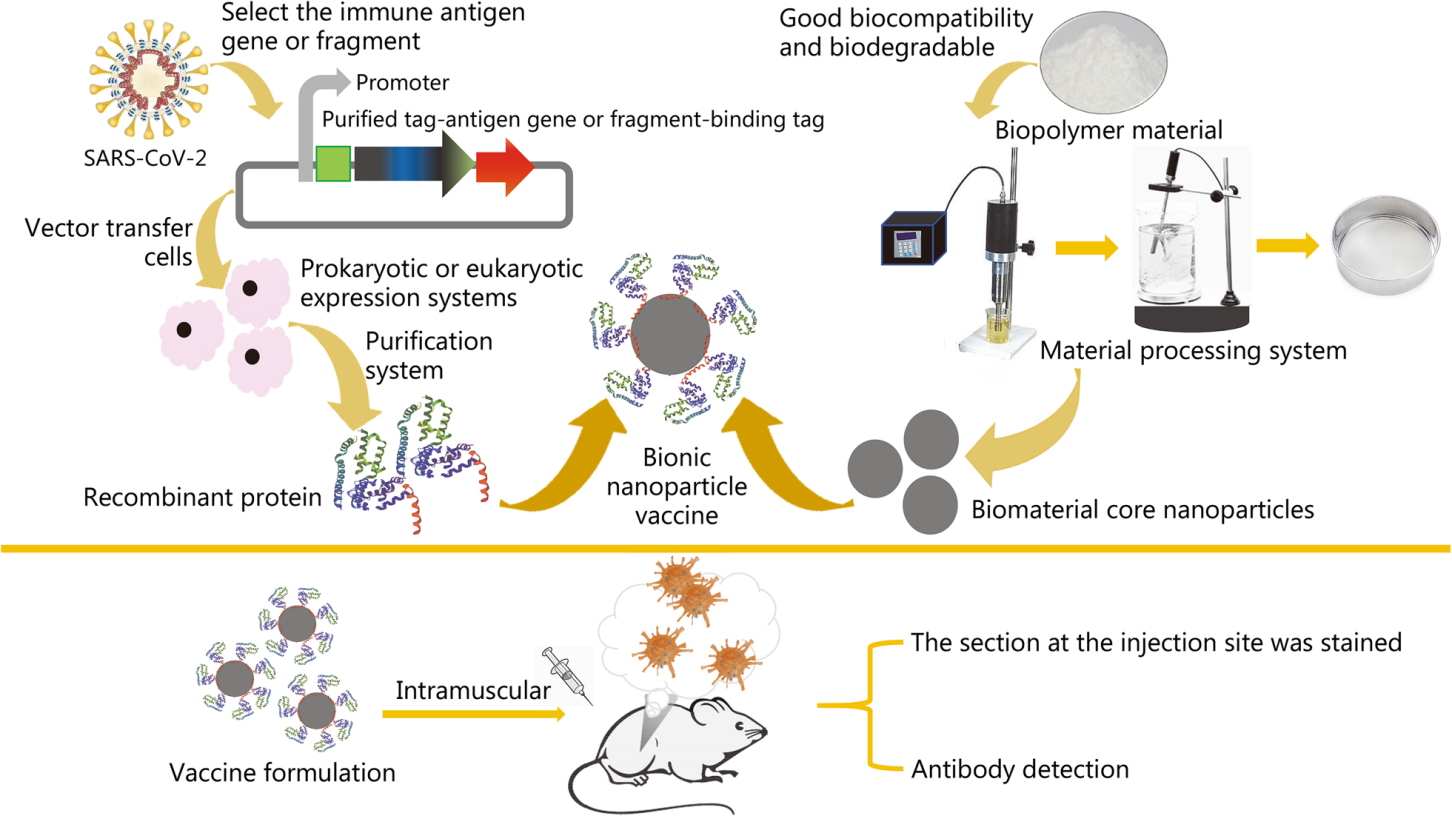
Schematic diagram of the preparation of the bionic nanoparticle vaccine

Bionic nanoparticle vaccines use biodegradable nanoparticles to replace the nucleic acid and proteins of the viral core, while their outer shell is decorated with recombinant viral surface proteins to form a virus-like structure through self-assembly. The surface of this virus-like spherical structure carries a large number of antigen molecules, which readily activates the immune response. Additionally, biomaterials stabilize the spherical structure, enabling it to remain intact inside the body while avoiding the degradation of surface proteins by related enzymes. Bionic nanoparticle vaccines are not infectious and have a defined composition with no viral nucleic acids, which provides excellent intrinsic safety and stability. In addition, the vaccine can concentrate viral antigen molecules and increase the protein content. Moreover, nanoparticles are more easily engulfed by immune cells, improving the efficiency of antigen presentation and resulting in rapid production of antibodies to neutralize the virus (Fig. 3f). Our team is in the process developing of this type of vaccine and we have obtained encouraging preliminary results (Fig. 4).

Many types of biomaterials can be used in this type of vaccine, including hydrophobic polymer materials represented by polylactic acid (PLA), poly (lactic-coglycolic acid) (PLGA), polycaprolactone (PCL), polyhydroxyalkanoates (PHAs, e.g., PHB, PBHV, PHBHHx, PHBVHHx, P34HB, etc.) and their derivatives [237–239]. Various biomaterials have different properties, but those used to prepare vaccine nanoparticles must be biocompatible and nontoxic. The most common material is PHA, which is a natural polyester stored inside cells as a source of energy and carbon. PHAs are produced by and accumulate in many bacteria and archaea under conditions of imbalanced nutrition (i.e., limited supply of nitrogen, oxygen or phosphorus, and excess carbon source) [240]. PHA monomers are characterized by diverse side chains and sequences, as well as chemical modifications. They have been used to synthesize polyesters with a variety of material properties. According to different requirements, the methods for producing and processing PHA in bacteria are different. However, inclusions that are purified are usually produced in bacteria. These inclusions are biocompatible and biodegradable and can be used in the fields of synthetic biology and biomedicine [241–243]. In addition, different ways to decorate biomaterials with viral proteins have been reported. Self-assembly of nanoparticles prepared by recombinant viral surface proteins containing a PhaP (binding protein of PHA or Phasing) tag and the hydrophobic polymer material PHA is one of the important approaches.

PhaP is a binding protein on the surface of PHA granules with good biocompatibility that is nontoxic. PhaP is an amphiphilic protein composed of four monomers with two conformations. It binds to the hydrophobic polymer material PHA through its hydrophobic binding site [244]. In addition, PhaP adheres to the surface of hydrophobic oil beads as a surfactant. Due to its widespread presence on the surface of PHA particles [245], PhaP is also the most widely applied protein among the four proteins on the surface of PHA [246].

The new COVID-19 vaccine strategy uses synthetic biology techniques combined with medical material-based nanoscience of previous vaccines. The focus of this strategy is to simulate the core–shell structure of the new coronavirus, which has unique advantages in activating the immune system. The whole structure is stable as a sphere and contains a large number of antigen molecules on the surface that enable it to efficiently activate the immune system and be easily recognized by antibodies in vivo, and thus it is engulfed by phagocytes. Scientists are using this strategy in the fields of immunity and tumors, and biomimetic simulation based on biomaterials is finding increasingly broad applications. However, its potential currently remains relatively underutilized in vaccine development.

Compared with inactivated natural viruses, biomimetic simulated self-assembled pseudovirus particles are safer, more efficient and more convenient. They are harmless to humans and do not require the operation of a biosafety level 3 or 4 laboratory. Therefore, biomimetic nanoparticles are more conducive to cytology research and animal model construction, as well as broad-based scientific research in general. Biomimetic pseudoviruses can be used for the development of bionic vaccines and therapeutic antigens, as well as for clinical treatment, which is very important for controlling viral epidemics. For COVID-19, this approach offers a rapid and efficient vaccine development strategy due to its high safety. At present, some teams have also conducted research on bionic nanoparticle vaccines [102, 247, 248].

Vaccines are the ultimate tools for the prevention and control of the COVID-19 pandemic. In the future, it will remain a hotspot of global attention.

## Conclusions

The World Health Organization (WHO) warns that SARS-CoV-2 may become a recurrent epidemic virus, and vaccines play a decisive role in overcoming epidemics. As of July 2021, the official website of the World Health Organization has listed more than 210 COVID-19 vaccines under development, more than 100 of which are in clinical development.

Although many teams worldwide are expending great efforts in the development of SARS-CoV-2 vaccines, risks and drawbacks are associated with the several vaccine types illustrated above. Potential biological safety problems have even been noted. A common problem is an insufficient ability to activate the immune system and poor immune effects. Reducing the risk of vaccines and improving their safety, efficiency and stability have become problems that urgently need to be solved. First, based on the functional mechanism of the COVID-19 vaccine inside the body, the viral protein acts as an antigen molecule, stimulating the human immune system and thereby inducing the production of antibodies to neutralize the virus. Therefore, scientists experience difficulties in delivering antigen molecules effectively and reducing the degradation of exogenous antigen molecules in the enzyme-rich environment of the body. Second, antigenic proteins can be obtained both directly from microorganisms and formulated in vitro or by delivering antigen-encoding genes into the body using various methods. Then, the host cells are used to synthesize antigen molecules, thereby activating the immune system and producing antibodies to neutralize viruses. In this case, scientists are faced with risks of integration into the host genome, producing in the worst case a transformed cell population that might replicate inside the body. As a result of various difficulties, the research and development of vaccines usually takes a long time. However, as countries around the world focus on tackling the problem, several vaccines are already on the market for human use (Tables 4 and 5).

Immunogenicity and duration of action are two important indices in the investigation of vaccines. At present, a variety of COVID-19 vaccines have entered clinical studies, and some vaccines have already completed Phase III/IV clinical trials (Table 5). Notably, the vaccines that have entered clinical trials will protect against newly emerged mutants of SARS-CoV-2. Recently, some studies showed, after the administration of one dose of vaccines (BNT162b2 or ChAdOx1 nCoV-19), noticeably lower effectiveness among persons infected with the delta variant [30.7% confidence interval (CI) and 95% CI 25.2–35.7] than among those with the alpha variant (48.7% CI; and 95% CI 45.5–51.7). For the BNT162b2 vaccine, the effectiveness of two doses was 93.7% (95% CI 91.6–95.3) among persons with the alpha variant and 88.0% (95% CI 85.3–90.1) among those with the delta variant. Similarly, for the ChAdOx1 nCoV-19 vaccine, the effectiveness of two doses was 74.5% (95% CI 68.4–79.4) among persons with the alpha variant and 67.0% (95% CI 61.3–71.8) among those with the delta variant [249]. In addition, the observed differences in the effectiveness of mRNA-1273 and BNT162b2 show that mRNA-1273 is almost twice as effective as BNT162b2 at protecting against the delta variant [250].

Currently, the hot topic of discussion also involves the patent of SARS-CoV-2 vaccines. Although we have applied for patent protection of the bionic nanoparticle vaccine in China, it is aimed at protecting this new vaccine strategy, not for commercial interests. Vaccine rolls out will take a long time. In other words, the bionic nanoparticle vaccine will not become economically viable for many years.

SARS-CoV-2 is prone to mutation, which imposes substantial challenges in the development of COVID-19 vaccines. Solving the problem of SARS-CoV-2 variants under the premise of ensuring safety poses a serious challenge to scientists. In the new bionic nanoparticle vaccine, all materials and their degradation products are presently found in the human body. Compared with the traditional types of vaccines, the main advantage of this biomimetic simulated virus vaccine is that the composition is clear and stable, and no potential biological safety problems exist. However, vaccines are not perfect, and different individuals experience different levels of side effects after vaccination. Some individuals experience no side effects, while others report some side effects. Bionic nanoparticle vaccines are also not immune to side effects. Of course, we are still concerned that an increased dose of bionic nanoparticle vaccines will also be needed to achieve the goal of improving immune protection, but this hypothesis has not been fully proven, and we will provide evidence in future studies. Among the characteristics of the biomimetic simulated virus vaccine, we focused on its structure to ensure that it is similar to the real virus with high efficiency and low risks. The slow degradation of the material may reduce the enzymatic damage to the antigen protein and achieve a lasting protective effect. Microspherical nanoparticles may serve as adjuvants to enhance their immunogenicity. This strategy might also shorten the development cycle and improve immune efficiency. Finally, its application prospects are also worth noting. Bionic simulation and nanoscience are brand new concepts, and thus a simulated virus vaccine is not currently on the market. We propose that this approach will have broad application prospects in vaccine development and research, antibody production, drug delivery and other aspects.

## Data Availability

Not applicable.

## Abbreviations

3CLpro: 3-Chymotrypsin-like protease
6HB: Six-helix bundle
ACE2: Angiotensin-converting enzyme 2
AD5-EBOV: Adenovirus type-5 vector-based Ebola vaccine
Ad5-nCoV: Adenovirus vector type 5
ADE: Antibody-dependent enhancement
ARDS: Acute respiratory distress syndrome
CEPI: Coalition for epidemic preparedness innovations
COVID-19: Coronavirus disease 2019
DENV: Dengue virus
ERVEBO (MSD): Ebola vaccine
FcγRIIa: Fc gamma receptor IIa
FDA: The US Food and Drug Administration
FP: Fusion peptide
HSV: Herpes simples virus
ICTV: International Committee on Taxonomy of Viruses
ID: Intradermal
IM: Intramuscular
ITRs: Inverted terminal repeats
IVI: Innovation and value initiative
KNIH: Korean National Institutes of Health
MERS-CoV: Middle East respiratory syndrome coronavirus
Mpro: Main protease
NSPs: Nonstructural proteins
NTD: N-terminal domain
NTD and CTD: N- and C-terminal domains
ORFs: Open reading frames
PCL: Polycaprolactone
PHA: Polyhydroxyalkanoate
PHEIC: Public Health Emergency of International Concern
PLA: Polylactic acid
PLGA: Poly(lactic-coglycolic acid)
PLpro: Papain-like protease
RBD: Receptor-binding domain
RdRp: RNA-dependent RNA polymerase
RSV: Respiratory syncytial virus
TMPRSS2: Transmembrane serine protease 2
SARS-CoV-1: Severe acute respiratory syndrome coronavirus 1
SARS-CoV-2: Severe acute respiratory syndrome coronavirus 2
UTRs: Untranslated terminal regions
VACV: Vaccinia virus
VLP: Virus-like particle
VOC: Variants of concern
VOI: Variants of interest
VSV: Vesicular stomatitis virus
WHO: World Health Organization
YF17D: Yellow fever virus 17D
